# Workflow for the Quantification of Soluble and Insoluble Carbohydrates in Soybean Seed

**DOI:** 10.3390/molecules25173806

**Published:** 2020-08-21

**Authors:** Ademar Moretti, Cintia L. Arias, Leandro A. Mozzoni, Pengyin Chen, Brant T. McNeece, M. A. Rouf Mian, Leah K. McHale, Ana P. Alonso

**Affiliations:** 1 BioDiscovery Institute, University of North Texas, Denton, TX 76201, USA; Ademar.Moretti@unt.edu (A.M.); Cintia.Arias@unt.edu (C.L.A.); 2 Crop, Soil and Environmental Sciences, University of Arkansas, Fayetteville, AR 72701, USA; lmozzon@uark.edu; 3 Fisher Delta Research Center, University of Missouri, Portageville, MO 63873, USA; Chenpe@missouri.edu; 4 USDA-ARS, Soybean & Nitrogen Fixation Unit, Raleigh, NC 27607, USA; brandon.mcneece@usda.gov (B.T.M.); rouf.mian@usda.gov (M.A.R.M.); 5 Department of Horticulture and Crop Science, The Ohio State University, Columbus, OH 43210, USA; mchale.21@osu.edu

**Keywords:** carbohydrates, soybean, oil, protein, soluble sugars, starch, matrix polysaccharide, crystalline cellulose, *Glycine max*

## Abstract

Soybean seed composition has a profound impact on its market value and commercial use as an important commodity. Increases in oil and protein content have been historically pursued by breeders and genetic engineers; consequently, rapid methods for their quantification are well established. The interest in complete carbohydrate profiles in mature seeds, on the other hand, has recently increased due to numerous attempts to redirect carbohydrates into oil and protein or to offer specialty seed with a specific sugar profile to meet animal nutritional requirements. In this work, a sequential protocol for quantifying reserve and structural carbohydrates in soybean seed was developed and validated. Through this procedure, the concentrations of soluble sugars, sugar alcohols, starch, hemicellulose, and crystalline cellulose can be determined in successive steps from the same starting material using colorimetric assays, LC–MS/MS, and GC–MS. The entire workflow was evaluated using internal standards to estimate the recovery efficiency. Finally, it was successfully applied to eight soybean genotypes harvested from two locations, and the resulting correlations of carbohydrate and oil or protein are presented. This methodology has the potential not only to guide soybean cultivar optimization processes but also to be expanded to other crops with only slight modifications.

## 1. Introduction

Soybean (*Glycine max* (L.) Merr.) is a commodity of great economic importance that annually raises billions of dollars in revenue for the main producer countries. The world soybean production reaches over 350 million metric tons per year and it is the fastest growing crop in terms of planted acres [1]. The main use of this legume is oriented toward the production of oil and soybean meal. The extracted oil may be refined for cooking and other edible uses or sold for biodiesel production or industrial applications. On the other hand, soybean meal is an important source of protein in animal diets, particularly for poultry, swine, cattle, fish, and other livestock [2,3]. The contents of oil and protein in soybean seed average approximately 20% and 40%, respectively. However, the precise composition depends on the plant’s genetics, environmental conditions, and management practices [4]. Breeders and genetic engineers have historically pursued increases in oil and protein [5,6,7], but, in recent years, research on carbohydrate content, composition, and utilization has aroused great interest.

At least three different approaches are under investigation to optimize the value added to soybean carbohydrates. The first one considers the development of specialty seed with a specific sugar profile to meet animal nutritional requirements. The main soluble carbohydrates present in soybean seeds are sucrose and the raffinose family of oligosaccharides (RFO), raffinose and stachyose. Parts of these carbohydrates remain in the soybean meal after oil extraction. However, while sucrose is a source of metabolizable energy, RFOs are considered antinutritional factors due to the lack of RFO-degrading enzymes in the digestive system of monogastric animals [8]. The removal of undesirable RFOs and the increment of sucrose content are critical for soybean value for the meal industry [8,9,10,11,12].

A second approach involves the redirection of carbons normally used for RFO synthesis to oil and/or protein, thus increasing their total concentration. During the last stages of seed maturation, there is a decrease in 10–15% of lipids that coincides with RFO accumulation and little or no maternal carbon supply. It has been hypothesized that carbon derived from the turnover of lipids and proteins contributes to the synthesis of RFOs. A recent study using fast neutron-mutagenized soybean populations with deletions in central carbon metabolic genes showed that a delayed switch in carbon allocation towards RFO biosynthesis resulted in extended lipid accumulation without compromising protein content [13].

The final approach is to target fibers. During soybean processing, a large number of carbohydrate-rich byproducts are generated, and finding valuable uses for them is highly desirable. Recent developments in their use as feedstocks for the production of biofuels, enzymes, and diverse specialty chemicals by microbial fermentation has been reported [14]. The case of soybean hulls is one of them. Hulls are separated from beans before oil extraction, representing almost 8–10% of the whole soybean, and around 18–20 million tons are being produced every year [15,16]. Moreover, among soybean byproducts, hulls are the preferred substrates for biofuel production because they contain minimal protein and high levels of cellulose and hemicellulose. However, the composition of the lignocellulose components affects the digestibility of the fibers. These fermentation processes using soybean carbohydrate as major feedstock to produce value-added bio-products have been an active area of research [14].

There are different methods currently available for carbohydrate quantification. Analytical methods applicable to routine industrial determinations or quality control are standardized in publications such as the “Official Methods of Analysis of AOAC International” or “ICC Standard Protocol” [17,18]. For research, some total carbohydrate assay kits are based on the phenol–sulfuric acid method, in which polysaccharides are hydrolyzed and the total monosaccharides are quantified by a colorimetric reaction (e.g., Abcam, RayBiotech, and Sigma Aldrich kits). However, compositional information on each reserve and structural carbohydrate requires method optimization and, often, more sophisticated techniques. Indeed, many procedures were developed to analyze one or two groups of compounds, e.g., oligosaccharide determination by high-performance liquid chromatography (HPLC) using a refraction index detector [19], quantification of sugars with high-performance anion-exchange chromatography with pulsed amperometric detection (HPAEC-PAD) [20,21], soluble sugar and starch extraction and quantification by enzymatic assays and HPLC [22], enzymatic extraction and quantification of starch [23], hemicellulose one-step acid hydrolysis and quantification by HPLC [24], and compositional analysis of plant cell wall hemicellulose and cellulose content using GC–MS and the anthrone method, respectively [25]. Nevertheless, the high variability within seeds [26] and the increasing number of studies related to carbohydrate relocation in soybean underpin the need for methods that allow the quantification of the different types of carbohydrates from the same sample. The aim of this work was to develop and validate a sequential protocol for extracting and quantifying soluble sugars (glucose, fructose, sucrose, maltose, verbascose, raffinose, and stachyose), sugar alcohols, starch, hemicellulose, and crystalline cellulose in soybean seed. The quantification procedures used colorimetric assays as well as particularly sensitive mass spectrometry detection (LC–MS/MS and GC–MS). The linearity, limit of detection (LOD), limit of quantitation (LOQ), accuracy, recovery, and matrix effect were determined. Finally, the workflow was applied to determine the carbohydrate composition in eight soybean genotypes harvested from two different locations. The main focus of this specific case study was to determine the degree to which seed carbohydrate content and composition vary depending on oil content and growing location. The data were analyzed using principal component analysis (PCA) and Pearson correlations [27,28] to find potential correlations with oil and protein content. Additionally, we anticipate that this workflow will facilitate cultivar optimization processes and the value addition of soybean carbohydrates.

## 2. Results and Discussion

### 2.1. Development of a Sequential Protocol for Quantifying Reserve and Structural Carbohydrates in Soybean Seeds

**Figure 1:**
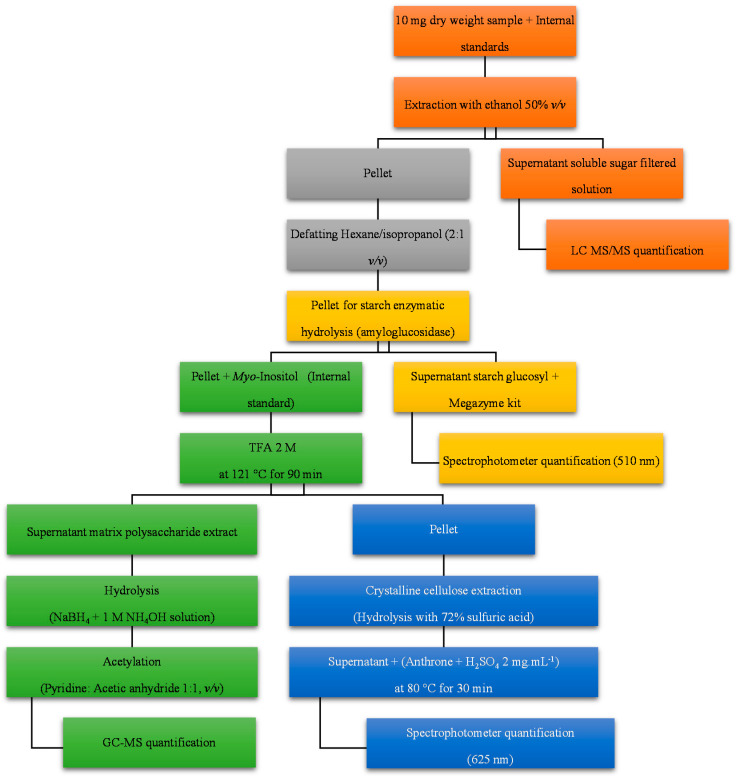
Scheme of the sequential carbohydrate extraction and quantification protocol. Each color refers to a specific part of the procedure. Soluble sugar and sugar alcohol extraction and quantification is shown in orange, oil removal in grey, starch extraction and quantification in yellow, hemicellulose extraction and quantification in green, and crystalline cellulose extraction and quantification in blue.

Carbohydrates, an underutilized major component of soybean seed, can be divided into soluble sugars, starch, hemicellulose, and crystalline cellulose. Their different structures and properties allow the development of a method whereby each species can be sequentially extracted from the same starting material. In Figure 1, an overview of all the steps applied for the extraction and quantification of carbohydrates in mature soybean seed is shown. This sequential protocol is a modified version of the one previously published [25], where the steps for extracting and quantifying soluble sugars and starch were added.

#### 2.1.1. Biological Sample Preparation

Soybean seeds are normally harvested with around 13% moisture. To be able to express an accurate quantity of carbohydrates per milligram of dry weight, soybean seeds must be totally dry. Therefore, the first step in the optimization of the sequential protocol was the evaluation of the drying process. The use of either a lyophilizer or an oven was evaluated with intact seeds or powder after grinding by a bead beater. Placing soybean powder into a forced circulation oven at 130 °C for one hour was the procedure that allowed more extensive removal of the moisture (Table S1). The amount of initial material was also tested, and the use of 10 mg dried seed powder resulted in the best recovery percentages (data not shown). Then, the whole workflow was optimized for a starting material consisting of 10 mg seed powder previously dried in a circulation oven at 130 °C for one hour.

#### 2.1.2. Soluble Sugars

There are different extraction and quantification methods available for soluble sugars. Xiaoli et al. determined that the optimal conditions for extraction of oligosaccharides in chickpea seeds, another legume with RFOs, were 50% (*v/v*) ethanol with 30 min incubation at 50 °C [19]. Using these conditions as a starting point, an extraction method for soluble sugars was tested whereby volumes and dried powder/solvent ratios were adapted to match soybean sugar content. The selected quantification method was LC-M/MS using multiple reaction monitoring mode, which allows the simultaneous detection of sugars, sugar alcohols, as well as ^13^C-labeled compounds that can be used as internal standards. For this reason, [U-^13^C_6_]-glucose and [U-^13^C_12_]-sucrose were added at the beginning of the extraction, directly to the dry powder, and used to account for treatment variability. It is important to note that the quantity of each sugar is very different, e.g., sucrose content in soybean is around 40 times in mass ratio and 100 times in molar ratio higher than verbascose [29]. In order to be able to quantify metabolites with such different ranges of concentration in a single run, MS parameters, especially sucrose declustering potential (DP), were optimized and [U-^13^C_12_]-sucrose was used to evaluate sucrose levels. The list of sugars and sugar alcohols quantified by this protocol as well as the selected transitions and MS parameters are presented in Table 1. An example of the chromatograms obtained using the external standard mix is presented in Figure S1.

From this list, three groups of sugar alcohols were not resolved chromatographically and coeluted when run as a mixture of metabolites: tetraols (erythritol and threitrol), pentitols (arabitol, ribitol and xylitol), and hexitols (galactitol and mannitol). The calibration curves of each soluble sugar and sugar alcohol showed strongly linear behavior, with correlation coefficients exceeding 0.98 in all instances (Table 1). The limits of detection (LOD) ranged between 0.3 nM for tetraols and 28 nM for glucose, and the limits of quantification (LOQ) between 0.9 nM and 93.5 nM (Table 1).

In order to further validate the LC–MS/MS quantification method, the matrix effect (ME) was evaluated for each soluble sugar and sugar alcohol. Except for tetraols and pentitols, the values for the ME were found to be within 100 ± 20% (Table 2), indicating that most of the metabolites analyzed did not show significant ion suppression or enhancement due to the matrix of the sample. The efficiency with which each compound was recovered from the biological sample was also determined. The recovery efficiency (RE) ranged between 82.80 and 115.34% (Table 2). Finally, an acceptable range of ± 20% the relative mean error (RME) was found for both intra-day and inter-day accuracy (Table 2).

#### 2.1.3. Starch

The second extraction step involves the hydrolysis of starch that remained in the insoluble pellet during soluble sugar extraction. After defatting the pellet, two strategies for starch enzymatic degradation were tested. The first one was described by Foster et al., where starch was hydrolyzed but not quantified [23]. The protocol used α-amylase and pullulanase, two glycosidases that can degrade starch α-1,4 and α-1,6-linkages, respectively. In the other method [30], amyloglucosidase was used on autoclaved pellets. This enzyme catalyzes the hydrolysis of terminal 1,4-linked α-glucose residues of starch. However, most forms of this enzyme can rapidly cleave 1,6-α-glycosidic bonds when the next bond in the sequence is 1,4-linked, as in the case of amylopectin [31]. After hydrolysis using either one of the procedures, glucose from starch was quantified by a colorimetric assay kit. The results showed that the enzyme pullulanase was contaminated with high levels of glucose (300 µg µL^−1^ enzyme). Although this is not specified in the information supplied by the provider, it is well known that glucose could be used in enzymatic formulations as it acts as a protein structure stabilizer [32]. Although the procedure developed by Foster et al. is acceptable for starch removal, it is not suitable for quantification [25]. Therefore, the method using amyloglucosidase on autoclaved pellets was chosen for starch hydrolysis [30].

Starch content in mature soybean seeds is very low (0.19–0.91%) [29,33]. Although the method for starch quantification that we included in this sequential protocol is not sensitive enough to quantify such low levels (minimum requirement of 1.4% of starch in the starting material), the extract obtained after the enzymatic hydrolysis of starch can be measured by LC–MS/MS using the same method described for soluble sugar quantification. We tested the colorimetric method in soybean embryos at different times during development, and we successfully followed starch accumulation with the Megazyme kit until the latest stages of maturation, when starch content decreases abruptly (unpublished results). Moreover, it was previously reported that, under elevated temperature and carbon dioxide concentrations, mature soybean seeds could contain 9–20% of their dry matter as starch [34], underlying other conditions under which the colorimetric method could be applied.

#### 2.1.4. Hemicellulose

After starch degradation, the pellet is mostly comprised of cell wall components: cellulose, hemicellulose, and polyphenol lignin. Hemicellulose can be selectively hydrolyzed with a weak acid such as trifluoroacetic acid (TFA). The monosaccharides derived from the matrix polysaccharide were further reduced and acetylated to their corresponding alditol acetate and quantified by GC–MS according to Foster et al. [25]. An example of the chromatogram obtained using the external standard mix is presented in Figure S2.

The GC–MS quantification method is extremely sensitive: the LOD and LOQ for each monosaccharide were calculated and ranged from 0.30 to 0.92 ng µL^−1^ and 1.10 to 3.06 ng µL^−1^, respectively (Table 3). Each calibration curve showed excellent linearity in the range of 3.33 to 333.33 ng µL^−1^ (R^2^ ≥ 0.9949).

In Table 4, the different parameters evaluated to validate the method are detailed. Both ME and RE were above 90%, demonstrating that there was no ion suppression from the biological matrix and that each monosaccharide was efficiently recovered. The intra- and inter-day accuracy was also assessed and the RME values were found to be within the ± 20% allowance range (Table 4).

#### 2.1.5. Crystalline Cellulose

The final step in the sequential extraction protocol comprises the hydrolysis of cellulose using concentrated sulfuric acid. Two procedures with differences in temperature and duration of the strong acid treatment were evaluated. Contrary to expectations, higher temperature and longer incubation time did not improve cellulose degradation (data not shown). Therefore, Foster’s protocol was used not only for cellulose chemical degradation but also for the derived glucose quantification by the anthrone method [25].

The linearity range was from 6.67 to 33.33 ng µL^−1^ of glucose, with a correlation coefficient of 0.9963, and the LOD and LOQ were 2.22 and 7.40 ng µL^−1^, respectively. The accuracy of the colorimetric method was within the acceptable range for intra-day and inter-day assays (Table 5).

### 2.2. Application of the Novel Sequential Method to Eight Soybean Genotypes Grown in Two Different Locations

#### 2.2.1. Carbohydrate Quantification in Eight Soybean Genotypes Grown in Two Different Locations

The workflow was successfully applied to eight soybean genotypes (G1-G8) harvested from two different locations: Arkansas (AR) and North Carolina (NC). In general, total soluble sugars (TSS), which includes the content of fructose, glucose, sucrose, raffinose, stachyose, verbascose, and maltose, extended between 101.22 and 140.36 mg g^−1^ (Table S3). These values are within the range reported for diverse soybean genotypes [11,35]. Sucrose and stachyose were found to be the main contributors to TSS; genotypes with up to 108.8 mg g^−1^ of sucrose and as little as 15.4 mg g^−1^ of stachyose were identified in this study (Table S3).

Previous studies in soybean seed revealed the existence of only minute amounts (around 0.001 to 0.03%) of sorbitol, arabitol, xylitol, and mannitol [36]. Others quantified sugar alcohols together with monosaccharides such as xylose [37], lacking sensitive methods to quantify them. The sensitivity and specificity of the LC–MS/MS method that we developed allowed us to quantify different groups of sugar alcohols (tetraols, pentitols, sorbitol, inositol, chiro-inositol, pinitol, galactinol, hexitols) which, when combined (TSA), ranged from 0.33 to 0.82% (Table S4).

Hemicellulose monosaccharide profiles of the eight genotypes grown in two locations are detailed in Table S5. The values of total matrix polysaccharide found were between 62.87 and 86.70 mg g^−1^. The more abundant sugar in the matrix polysaccharide was galactose (28.4 mg g^−1^ average), followed by arabinose (14.0 mg g^−1^ average) and mannose (12.5 mg g^−1^ average). The content of glucose in our results is lower than the values reported in other studies, where methods with lesser control in the weak hydrolysis of cell wall components could lead to higher levels of glucose in the hemicellulose fraction due to partial hydrolysis of crystalline cellulose [38,39,40].

In the different soybean genotypes, glucose derived from cellulose ranged between 3.45 and 5.22% (Table S6). Cell wall analyses performed on mature soybean seeds often report neutral detergent fiber that includes lignin, cellulose and hemicellulose, where the seed coat is the major contributor [41]. Considering that seeds contain 13% neutral detergent fiber, (6.3–8.7%) hemicellulose, and around 0.5% lignin [42,43], the average of 4.3% of cellulose obtained in our lines is within the expected range.

Besides carbohydrates content, oil and protein levels were measured, as described in the Materials and Methods. The ranges of oil and protein content were found to be 22.01–26.21% and 37.18–43.46%, respectively (Table S6).

#### 2.2.2. Line Variability and Location Effect

When the information of the eight genotypes grown in two locations (Arkansas, AR, and North Carolina, NC) was displayed as principal component analysis (PCA), no separation was evident between genotypes or locations when considering the first five components that explained 73.1% of the total variance (Figure S3). The most informative component, PC1, explained the largest part of the data variance (31.3%), while PC2 and PC3 explained 13.6 and 11.7%, respectively. It can be observed that sugar alcohols, especially galactinol, pinitol, inositol, and TSA, had the opposite effect on PC1 to the hemicellulose components, particularly rhamnose, fucose, galactose, and THC (Figure S4, Table S2). On the other hand, PC2 was positively affected by pentitols, hexitols, and arabinose (Figure S4, Table S2). From the first five components, PC3 is the most influenced by protein and oil and, interestingly, the content of hexitols and maltose modified the component in the same direction as oil but opposite to protein (Figure S4, Table S2).

The variability of carbohydrate, oil, and protein content between genotypes grown in the same location was also evaluated. Ten features presented significant differences between genotypes in each location (Table 6).

**Figure 2:**
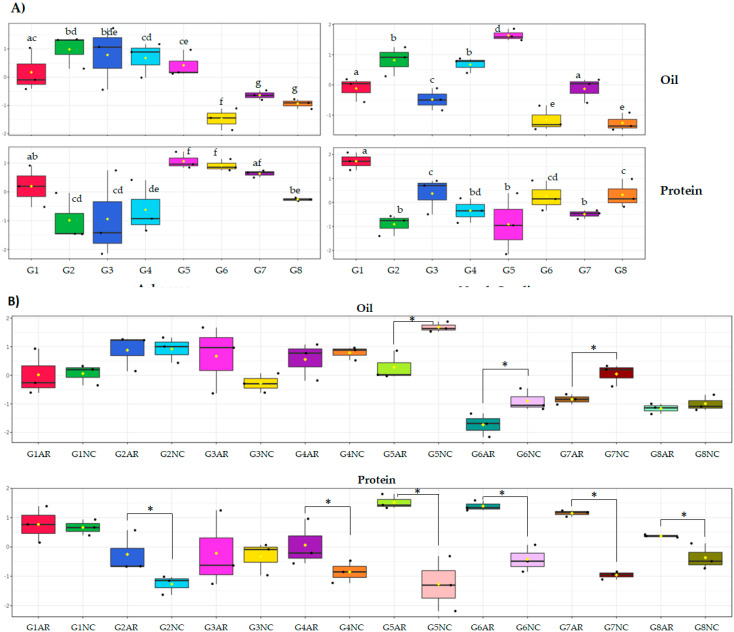
Oil and protein content in the eight soybean genotypes (G1 to G8) cultivated in two different locations (Arkansas, AR, and North Carolina, NC). The percentages were log-transformed and auto-scaled using MetaboAnalyst software for better visualization. (**A**) Comparison at each location and (**B**) comparison across locations. The differences in oil content between locations of the three genotypes that were significantly different ranged between 0.9 and 1.8%, while the differences in protein content between locations of the six genotypes that were significantly different ranged between 1.6 and 6.3%. Significant differences are noted with letters or asterisks in A and B, respectively (ANOVA, Fisher LSD, *p* < 0.05, n = 3).

It is worth mentioning that the concentrations of oil and protein not only presented differences between the genotypes in the same location (Figure 2A) but also, as it was expected, in the same genotypes between regions (Figure 2B). For instance, when genotype G5 was grown in AR, it showed one of the highest levels of protein and an average oil content; however, when cultivated in NC, it was one of the lowest in protein and the highest in oil (Figure 2). Evidently, there was a GxE effect on protein and oil concentrations due to environmental factors across locations.

**Figure 3:**
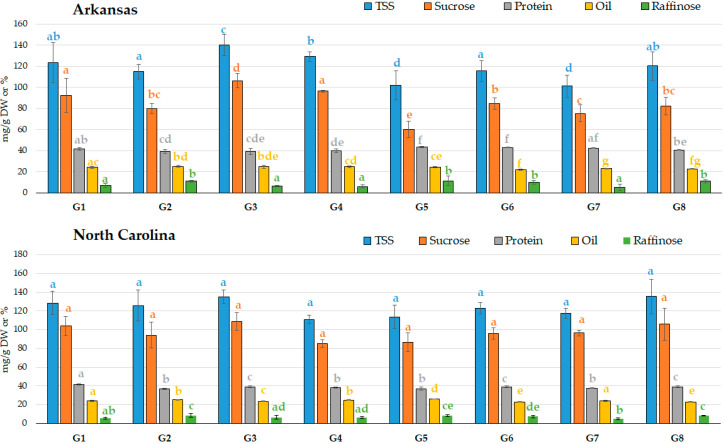
Total soluble sugars, sucrose, oil, protein, and raffinose content in the eight soybean genotypes (G1 to G8) grown in two different locations (Arkansas and North Carolina). Total soluble sugars (TSS), sucrose, and raffinose are presented in mg g^−1^ DW, while protein and oil are expressed as percentages. Significant differences between genotypes per location are indicated by different letters (ANOVA, Fisher LSD, *p* < 0.05, n = 3).

Moreover, while the carbohydrate differences were centered in soluble sugars and sugar alcohols between the genotypes grown in AR, the genotypes grown in NC presented more differences in the hemicellulose composition (Table 6). In Figure 3, five of these features (oil, protein, TSS, sucrose, and raffinose) are presented for both locations as an example. It is interesting to note that the genotype G3 grown in AR showed high TSS, sucrose, and oil, average protein content, and one of the lowest levels of raffinose; these characteristics were closely maintained when grown in NC (Figure 3).

**Figure 4:**
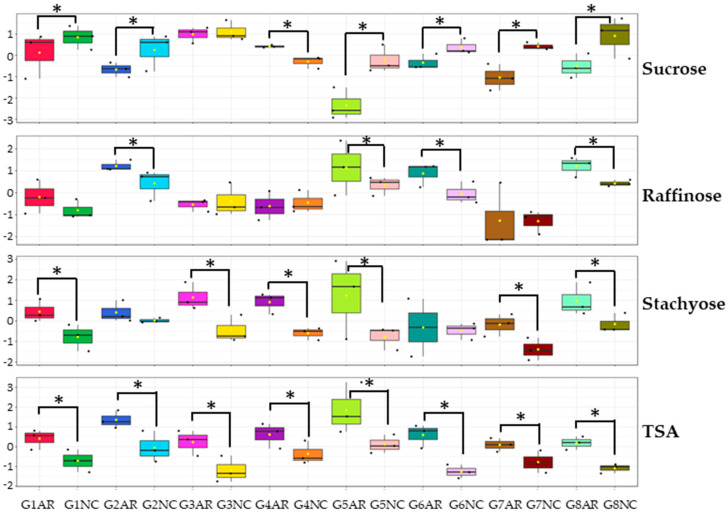
Main soluble sugar and total sugar alcohol content in the eight soybean genotypes (G1 to G8) cultivated in two different locations (Arkansas, AR, and North Carolina, NC). The content levels in mg were log-transformed and auto-scaled using MetaboAnalyst software for better visualization. The comparison was done across locations for each genotype. Significant differences are noted with asterisks (ANOVA, Fisher LSD, *p* < 0.05, n = 3).

As observed for protein and oil, the carbohydrate content also showed differences when comparing the same genotype grown at each location. In Figure 4, an example of this comparison is presented for the key soluble sugars (sucrose, raffinose, and stachyose) and the highly variable TSA content. For the genotypes that showed significant differences in the content of raffinose and stachyose, the level of these oligosaccharides was always higher in AR (Figure 4). The same effect was observed for the TSA content that was affected in the eight genotypes: in each case, the seeds harvested in AR had higher levels than in NC (Figure 4). However, sucrose showed the opposite trend, with higher levels in seeds harvested in NC, with the exception of the genotype G3, which did not show a significant difference, and G4, which presented a higher level of this disaccharide in AR (Figure 4). This result suggests better environmental conditions in NC, considering the desired high sucrose/low RFO phenotype searched with no strong dependence in the genotype.

**Figure 5:**
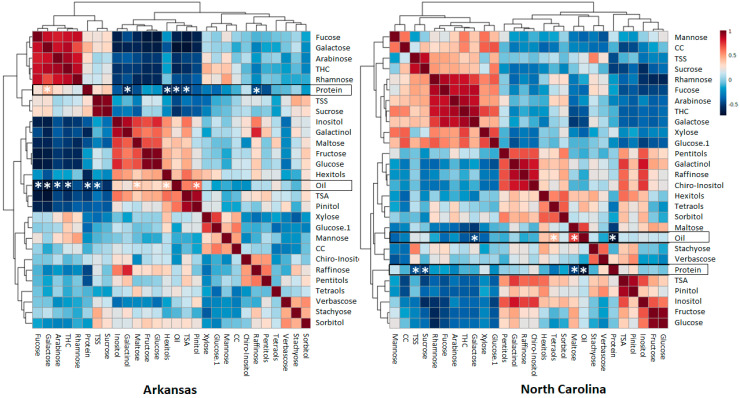
Pearson’s correlation analysis. The analysis was performed separately for each location. Metabolites that significantly correlate with oil and protein are indicated with a white star (*p* < 0.05, n = 3). Glucose.1: glucose from matrix polysaccharide, TSS: total soluble sugars, TSA: total sugar alcohols, THC: hemicellulose components, CC: crystalline cellulose.

Pearson correlation analysis was also performed for each location (Figure 5). Despite the well-known negative correlation between oil and protein [13] that was observed in both locations (r ≤ −0.51, *p* < 0.05), new correlations were identified. In agreement with PC3 loading coefficients (Figure S4, Table S2), although maltose content is low in comparison to the other sugars (Table S3), it showed a significant positive correlation with oil in both locations (r ≥ 0.42, *p* < 0.05) and a negative correlation with protein that was significant in AR (r ≤ −0.48, *p* < 0.05), with the same tendency in NC (r ≤ −0.4, *p* < 0.1, Figure 5). On the other hand, raffinose and its precursor galactinol showed a strong negative correlation with protein content that was significant only in NC (r ≤ −0.42, *p* < 0.05), with a similar trend in AR. Interestingly, the enzyme that synthesizes galactinol, galactinol synthase, was proposed as an important regulator of carbon partitioning in developing soybean seeds [44]. In addition, the concentration of galactose in hemicellulose also correlated negatively with oil (r ≤ −0.46, *p* < 0.05) in both locations and positively with protein in NC (r ≥ 0.45, *p* < 0.05). Finally, hexitols and tetraols correlated positively with oil in at least one location (r ≥ 0.42, *p* < 0.05), and hexitols were also found to correlate negatively with protein in NC (r = −0.53, *p* < 0.05, Figure 5).

## 3. Materials and Methods

### 3.1. Reagents, Chemicals, and Enzymes

[U-^13^C_6_]-glucose and [U-^13^C_12_]-sucrose were ordered from Cambridge Isotope Laboratories, Inc (Tewksbury, MA, USA). Unlabeled mono-, di-, oligosaccharide, and sugar alcohol standards were purchased from Millipore-Sigma (St. Louis, MO, USA), as well as the pullulanase microbial enzyme. On the other hand, α-amylase and amyloglucosidase were part of the Total Starch Assay Kit from Megazyme (Wicklow, Ireland). LC–MS grade acetonitrile, ethyl acetate, and acetone were obtained from Thermo Fisher Scientific (Waltham, MA, USA). Except when otherwise noted, all the chemicals used were analytical grade.

### 3.2. Standards Preparation

Carbohydrate external and internal standards were reconstituted in 100% ultrapure water. [U-^13^C_6_]-glucose and [U-^13^C_12_]-sucrose were prepared as a mix solution with a concentration of 2.5 mM and 0.5 mM, respectively. The stock concentration of *Myo*-Inositol standard was 5 mg mL^−1^.

The standard curves were generated through serial dilutions of each metabolite, using up to six points. The linearity range was delimited by the lowest and highest concentration evaluated in the construction of the curve that followed a linear behavior with the variable measured. For LC–MS/MS and GC–MS quantification, the limits of detection (LOD) and quantification (LOQ) were defined as three and ten times the signal-to-noise ratio, respectively. For the colorimetric assays, they were defined as three and ten times the ratio between the standard deviation of y-axis intercept and the slope of the calibration curve [45].

The external standard mix, run in parallel to the samples for LC–MS/MS quantification, consisted of 100 µM verbascose, 10 µM maltose, 50 µM of each pentitol and hexitol, and 1 µM of the rest of the sugars and sugar alcohols quantified (Table 1). On the other hand, for GC–MS quantification, the external standard mix solution contained 5 mg mL^−1^ of each monosaccharide quantified (Table 3).

### 3.3. Plant Growing Locations and Conditions

Soybean genotypes (Table S3) denoted by “N16” are F4:8 genotypes (breeding lines) developed by the USDA-ARS in Raleigh, North Carolina. Soybean genotypes denoted by the prefix “R” are F4- or F5-derived breeding lines developed by the University of Arkansas in Fayetteville, AR. NC-Dunphy was a conventional check cultivar used in the trial which was previously released by North Carolina State University. AG 56X8 and AG 59X7 were commercial checks.

The North Carolina trial was conducted at the Central Crops Research Station in Clayton, NC. Each genotype consisted of three-row plots replicated in triplicate in a randomized complete block design. Planted plot length was 5.8 m, with seed density of 26 seeds m^−1^. Before harvest, plots were end-trimmed to a length of 4.6 m. Only the center row of the plots was harvested at maturity using a plot combine. Plot combines were cleaned with forced air prior to harvesting the next plot.

In Arkansas, the trials were planted at the Rice Research and Extension Center near Stuttgart, AR. Plots consisted of two rows, 5.8 m long and 0.75 m apart, with a seed density of 26 seeds m^−1^. Plots were machine-harvested at maturity.

### 3.4. Seed Oil and Protein Quantification

Seeds from the harvested plots were visually inspected and cleaned to remove off-types and debris in order to ensure quality assessment of each genotype. An 80 g subsample of the cleaned seed was then analyzed for seed oil and protein content using a Perten DA 7250 Near Infrared Reflectance (NIR) Analyzer (Perten Instruments^®^, Hägersten, Sweden) and reported on a zero-moisture basis.

### 3.5. Carbohydrate Sequential Extraction

#### 3.5.1. Biological Sample Preparation

The first step in the optimization of the sequential extraction protocol was the evaluation of the procedure to remove the seed moisture. Either the use of a lyophilizer for three days or a forced circulation oven at 130 °C for one hour [46] was tested on intact seeds or powder after grinding. For grinding, four soybean seeds were used in a 10 mL mill grinding jar using a bead beater for 5 min at 30 Hz (Restch MM 400, Haan, Germany). The protocol was further evaluated with 10 mg starting material.

#### 3.5.2. Soluble Sugar and Sugar Alcohol Extraction

A modified version of the protocol published by Xiaoli et al. was used to extract soluble sugar and sugar alcohol from soybean seed powder [19]. In order to correct for treatment variability, first, 10 µL of a mix solution of 2.5 mM of [U-^13^C_6_]-glucose and 0.5 mM of [U-^13^C_12_]-sucrose was added to the samples as an internal standard. Two separate tubes containing only the internal standards were treated in parallel to the samples. Three consecutives soluble sugar extractions with 0.5 mL of 50% (*v/v*) ethanol at room temperature were performed. Each extraction consisted of 5 min agitation at 30 Hz using a bead beater, 30 min incubation at 50 °C in a water bath, and centrifugation 10 min at 17,000× *g*. From the pooled supernatant (1.5 mL total volume), 0.5 mL was then filtered with a 0.2 µm Nanosep centrifugal device (New York, NY, USA) for 15 min at 17,000× *g* at room temperature. This filtrate was used for soluble sugar and sugar alcohol quantification by LC–MS/MS.

#### 3.5.3. Defatting

The remaining pellet after soluble sugar extraction was defatted by the addition of 1.5 mL of hexanes/isopropanol (2:1, *v/v*) and agitation for 5 min at 30 Hz. Then, the samples were centrifuged for 15 min at 17,000× *g* at room temperature, and the supernatant was discarded. After oil removal, the pellets were washed with 1 mL of water, centrifuged for 15 min at 17,000× *g* at room temperature, and the supernatant was discarded. The remaining pellets were saved for starch extraction.

#### 3.5.4. Starch Extraction

Two methods for starch degradation were evaluated. The first one, described by Cocuron et al., consists of the incubation of previously autoclaved pellets with amyloglucosidase in 0.1 M acetate buffer pH 4.8 for 2 h at 55 °C [30]. The second procedure, described by Foster et al., uses α-amylase and pullulanase, with overnight incubation at 37 °C in a shaker instead [25]. In both cases, a 0.5 mL aliquot of the degraded starch (glucose units) was used for quantification. As the commercial pullulanase was discovered to be contaminated with high levels of glucose, not reported by the provider, the first starch extraction procedure using amyloglucosidase was the method of choice for starch quantification. Finally, the pellets were washed with 1.5 mL of water and centrifuged for 15 min at 17,000× *g* at room temperature. The supernatant was discarded and 500 μL of acetone was added, vortexed, and dried under a stream of nitrogen.

#### 3.5.5. Hemicellulose Extraction

Hemicellulose components were extracted according to Foster et al., with some modifications [25]. First, 60 µL of a 5 mg mL^−1^ *Myo*-Inositol solution was added as an internal standard; then, 250 µL of acetone was added, and the pellet was dried under nitrogen flow. Two separate tubes containing only the internal standards were treated in parallel to the samples. The weak acid hydrolysis of the pellet was conducted with 750 µL of 2 M trifluoroacetic acid (TFA), incubated for 90 min at 121 °C in a heating block. After cooling in ice, the samples were centrifuged at 17,000× *g* for 15 min, and 100 µL of the acidic supernatant containing the matrix polysaccharide-derived monosaccharides was transferred to a new tube. The TFA was evaporated under nitrogen flow at 40 °C. Finally, 600 µL of 2-propanol was added to each sample, followed by agitation and solvent evaporation under nitrogen flow at 40 °C. These dried samples were later used for alditol acetate derivatization and GC–MS quantification of the hemicellulose components. The pellet remaining after hemicellulose extraction was saved for crystalline cellulose analysis.

#### 3.5.6. Cellulose Extraction

Two procedures were evaluated for crystalline cellulose hydrolysis. In the first one, described by Foster et al., one milliliter of a solution of acetic acid: nitric acid: water (8:1:2, *v/v/v*) was added to the pellet remaining after hemicellulose extraction, and the mixture then incubated in a heating block at 100 °C for 30 min [25]. The samples were cooled on ice until reaching room temperature and then centrifuged at 17,000× *g* for 15 min. The supernatant was discarded and the remaining material was washed with 1.5 mL water and 1.5 mL of acetone. For each wash, after the addition of the solvent, the samples were centrifuged for 15 min at 17,000× *g* at room temperature, and the supernatant was discarded. After this treatment, only cellulose remained insoluble in the pellet. The last hydrolyzing treatment was performed within 175 µL of 72% sulfuric acid solution at room temperature for 30 min; the mixture was vortexed and incubated for 15 min more. In the second protocol [47], stronger incubation conditions were applied (100 °C for 3 h). However, this latest procedure was less effective for cellulose degradation (data not shown), so Foster’s method was chosen for our analyses [25]. Finally, 825 μL of water was added; then, the samples were vortexed and centrifuged at 17,000× *g* for 15 min. The supernatant was used for glucose quantification by the colorimetric anthrone assay.

### 3.6. Carbohydrate Quantification

#### 3.6.1. Soluble Sugar and Sugar Alcohol Quantification by LC–MS/MS

For quantification, 50 μL of the filtered extract (Section 3.5.2) was diluted with 950 μL of acetonitrile/water (60:40, *v/v*), and 5 μL was injected into a 2.0 × 150 mm Shodex Asahipak NH2P-50 2D column with a Shodex Asahipak NH2P-50G 2A guard column (Showa Denko America, New York, NY, USA). The separation was performed with an ultrahigh-pressure liquid chromatography (UHPLC) 1290 Infinity II from Agilent Technologies (Santa Clara, CA, USA). The mobile phase was a gradient with acetonitrile/water (A/B) at a constant flow of 0.4 mL.min^−1^, following the scheme 0–8.5 min 85% A, 8.5–15 min 78% A, 15–19 min 72% A, 19–25 min 68% A, 25–30 min 85% A. The MS/MS analyses were performed with a linear triple-quadrupole/ion trap mass spectrometer QTRAP 6500+ (AB Sciex Instruments, Framingham, MA, USA). The different metabolites were simultaneously detected using multiple reaction monitoring (MRM) in negative mode. The source parameters used were curtain gas (30 psi), temperature (350 °C), nebulizer gas (60 psi), heating gas (60 psi), and collision activated dissociation (Medium) were kept constant during the MRM. Mass spectra were acquired using ionization exchange by negative electrospray (−4500 V), with a dwell time of 150 msec. The transitions, retention times, and parameters used for each metabolite are detailed in Table 1. LC–MS/MS data were acquired and processed using Analyst 1.7.0 software (AB Sciex Instruments, Framingham, MA, USA). The quantification was accomplished using i) [U-^13^C_6_]-glucose and [U-^13^C_12_]-sucrose as internal standards to account for any loss of material during sample preparation; ii) unlabeled mono-, di-, oligosaccharide, and sugar alcohol external standards with known concentrations.

#### 3.6.2. Glucose from Starch Quantification by a Colorimetric Assay

Glucose from starch was quantified by the method of glucose oxidase/peroxidase (GOPOD) from Total Starch Assay Kit (Megazyme, Wicklow, Ireland). Briefly, 35 μL of starch extract was mixed with 1 mL of the GOPOD reagent, incubated for 20 min at 50 °C, and absorbance at 510 nm was measured.

#### 3.6.3. Derivatization of Hemicellulose Components and Quantification by GC–MS

The procedure was performed according to Foster et al., with minor modifications [25]. Prior to the quantification, two derivatization steps are needed. The first involves reduction of the monosaccharides to their corresponding alditols and then their acetylation. For this, 200 µL of a sodium borohydride solution was added to each dried sample and then incubated for 1.5 h at room temperature. The reaction was neutralized by the addition of 150 µL glacial acetate, followed by agitation and solvent evaporation under nitrogen stream at 40 °C. The dried acetates were washed with 500 µL methanol 100%, agitated and dried under nitrogen. To conclude the derivatization process, 50 μL of acetic anhydride and 50 μL of pyridine were added, followed by agitation. Then, samples were incubated at 121 °C for 20 min in a heating block, allowed to cool in ice, and then dried under nitrogen gas at 40 °C. Next, the alditol acetates were re-suspended with 600 µL of toluene, followed by the last drying step. To finally extract the alditol acetates, 500 µL ethyl acetate and 2 mL of Milli-Q water were added, stirred, and centrifuged at 750× *g* for 5 min. In total, 50 µL of the supernatant was then diluted with 100 µL of acetone and the dilution was used for GC–MS quantification. The samples were analyzed in a Trace 1310 gas chromatograph coupled to an ISQ single-quadrupole mass spectrometer from Thermo Fisher Scientific (Waltham, MA, USA). Electron impact ionization (EI) was used with range 50–600 amu, transfer line, and an ion source temperature of 280 °C. A TriPlus RSH autosampler was used to automatically inject the samples; the injection volume was set to 1 μL with a split 10, and the injector temperature was 260 °C. The separation was performed by a Supelco SP-2380 (Bellefonte, PA, USA) (30 mm × 0.25 mm × 0.25 μm film thickness) column with an 8 min solvent delay and a flow rate of 1.5 mL min^−1^, using helium as carrier gas. The temperature program was as follows: initial hold at 160 °C for 2 min; a 20 °C min^−1^ ramp to 200 °C and hold for 5 min; a 20 °C min^−1^ ramp to 245 °C and hold 10.5 min, a 100 °C min^−1^ ramp to 270 °C and hold 3 min; spike to 270 °C and hold for 5 min before cooling to the initial temperature of 160 °C. GC–MS data were acquired and processed using XCalibur 4.4 software (Thermo Fisher Scientific, Waltham, MA, USA), and the NIST17 library was used for mass spectra confirmation of each metabolite. The quantification was accomplished using (i) *Myo-* inositol as the internal standard to account for any loss of material during sample preparation; (ii) monosaccharide external standards with known concentrations.

#### 3.6.4. Cellulose-Derived Glucose Quantification by the Anthrone Method

The glucose content obtained from cellulose hydrolysis was quantified by a colorimetric assay according to Foster et al. [25]. For the anthrone method [48], 90 μL water and 200 µL anthrone dissolved in concentrated sulfuric acid (2 mg anthrone per mL sulfuric acid, 99%) was added to 10 µL of extracted cellulose. The samples were incubated at 80 °C for 30 min. The absorbance of samples at 625 nm was measured using a Synergy MX microplate reader spectrophotometer (BioTek Instruments, Inc., VT, USA).

### 3.7. Determination of Accuracy, Recovery, and Matrix Effect

#### 3.7.1. Accuracy

The accuracy was assessed by studying repeatability (intra-day) and intermediate accuracy (inter-day). For LC–MS/MS and GC–MS quantifications, the repeatability was determined for each metabolite using four soybean biological samples and three different concentrations of a mixture of external standards; each concentration was evaluated by four biological replications. The standard mix contained all metabolites quantified by the technique, and the concentrations used were 0.25, 0.50, and 1.00 µM for soluble sugars/sugar alcohols and 25, 50, and 100 µg for hemicellulose components measured by LC–MS/MS and GC–MS, respectively. Samples with no addition of standard mix were used to account for the metabolite concentration present in the biological samples. The intermediate accuracy was obtained by analyzing the spiked samples on three different days by four biological replications. The accuracy was determined as the relative mean error (RME) in percentage [49] and was calculated as follows: RME (%) = 100 × [peak area (sample spiked after extraction) − peak area (sample)]/mean peak area (external standard).

On the other hand, for the crystalline cellulose quantification method, the analysis was performed using five different concentrations of glucose standard, each by four biological replications, and measured either on the same day for intra-day accuracy or on three different days for inter-day accuracy. The accuracy was determined by calculating the RME, as previously described.

#### 3.7.2. Recovery

The recovery analysis was carried out for soluble sugars and hemicellulose extracts. It was calculated as previously described [30], using the following equation: recovery (%) = 100 × [analyte peak area (sample spiked before extraction) − analyte peak area (sample)]/[analyte peak area (sample spiked after extraction) − analyte peak area (samples)].

#### 3.7.3. Matrix Effect

The matrix effect (ME) is generated through interactions between the analyte and the matrix coextractive. The analysis of the matrix interaction was carried out for soluble sugars and hemicellulose quantifications. The matrix effect was evaluated following the published procedure [50] that uses the following equation: ME (%) = 100 × [analyte peak area (sample spiked after extraction) − analyte peak area (sample)]/average analyte peak area (external standard).

### 3.8. Statistical Analysis

Principal component analysis (PCA), ANOVA, Fisher LSD, and Pearson correlation analysis were performed (*p* < 0.05) on log-transformed data using the free web-based statistical software MetaboAnalyst 4.0 [51]. For the PCA, principal components, data scores, and loading coefficients were determined too. For all these analyses, three biological replicates were used for carbohydrates, oil, and protein.

## 4. Conclusions

In this work, a sequential protocol to extract and quantify soybean carbohydrates was developed and validated. The novelty of this workflow relies on its ability to use the differences in structural and solubility properties of the carbohydrate classes to allow consecutive extractions in which each step does not affect the following ones. Consequently, it presents the unique advantage of quantifying a total of 23 compounds or groups of compounds with high accuracy and sensitivity as part of soluble sugars, sugar alcohols, starch, hemicellulose matrix components, and crystalline cellulose. The approximated throughput is 40 samples per week for the entire workflow. This method was applied to determine the carbohydrate composition of eight soybean genotypes grown in two different locations and study correlations with oil and protein, which is important to guide future breeding strategies. Although this technical advance was directed toward soybean seed, it is fully applicable to other organisms and tissues, with minor modifications. Furthermore, we anticipate that this novel approach will contribute to the ongoing research of soybean carbohydrate utilization and facilitate cultivar optimization processes. Finally, this protocol opens new avenues for carbohydrate measurement in other industries, such as the pharmacological and cosmetic analytical chemistry fields [52,53,54].

## Figures and Tables

**Table 1:** Sensitivity and linearity of LC–MS/MS quantification method for soluble sugars and sugar alcohols.

Sugars and Sugar Alcohols	Transition (*m/z*)	RT	DP	CE	CXP	Linearity Range (nM)	R^2^	LOD	LOQ
		(min)	(Volts)	(Volts)	(Volts)			(nM)	(nM)
Erythritol	121.1/89.0	2.8	–20	–13	–11	200–100,000	0.9927	0.3	0.9
Threitol	121.1/89.0	2.8	−20	−13	−11	200–100,000	0.9903	0.3	1.0
Arabitol	150.9/89.0	3.8	−40	−16	−37	200–100,000	0.9956	6.0	20.0
Ribitol	150.9/89.0	3.8	−40	−16	−37	200–100,000	0.9923	6.1	20.3
Xylitol	150.9/89.0	3.8	−40	−16	−37	200–100,000	0.9993	11.2	37.4
Pinitol	193.0/161.0	4.4	−35	−14	−9	20–100,000	0.9942	0.7	2.4
Fructose	179.0/89.0	4.9	−40	−10	−9	200–100,000	0.9985	14.9	49.6
Sorbitol	181.0/89.0	6.1	−50	−20	−9	200–100,000	0.9992	3.6	11.9
Mannitol	181.0/89.0	6.8	−50	−20	−9	200–100,000	0.9815	2.6	8.6
Galactitol	181.0/89.0	6.8	−50	−20	−9	200–200,000	0.9965	5.6	18.7
Glucose	179.0/89.0	7.7	−40	−10	−9	2000–200,000	0.9902	28.0	93.5
*Chiro*-Inositol	179.0/161.0	10.8	−40	−16	−27	20–100,000	0.9911	4.1	13.6
Inositol	178.9/87.0	11.1	−65	−22	−9	20–10,000	0.9940	0.5	1.7
Sucrose	341.1/89.0	11.9	−200	−23	−21	20–100,000	0.9974	4.5	15.0
Maltose	341.1/161.0	13.3	−50	−10	−7	200–100,000	0.9982	2.7	8.8
Galactinol	341.0/179.0	17.2	−120	−22	−1	20–200,000	0.9999	4.6	15.3
Raffinose	503.2/178.9	17.4	−140	−32	−11	200–100,000	0.9976	1.0	3.2
Stachyose	665.1/383.1	21.2	−220	−48	−25	200–200,000	0.9996	0.5	1.6
Verbascose	828.1/545.1	23.9	−280	−52	−25	2000–500,000	0.9786	21.1	70.2

Limits of detection (LOD) and limits of quantification (LOQ) were obtained based on a signal-to-noise ratio of 3:1 and 10:1, respectively. The entrance potential (EP) was −10 V in all instances. RT: retention time, DP: declustering potential, CE: collision energy, CXP: collision cell exit potential, R^2^: correlation coefficient. An injection volume of 1 µL was considered.

**Table 2:** Recovery (%), matrix effect (%), intra- and interday accuracy (%) of the soluble sugars and sugar alcohols.

				Accuracy (%)					
				Intra-Day Assay (n = 4)			Inter-Day Assay (n = 12)		
	Carbo-hydrates	RE (%)	ME (%)	0.25 µM *	0.50 µM *	1.00 µM *	0.25 µM *	0.50 µM *	1.00 µM *
Soluble Sugars	Fructose	99.39	100.46	−0.99	−0.96	−0.94	−0.99	−0.97	−0.95
	Sucrose	95.26	109.57	−1.65	1.01	1.64	−0.58	1.90	3.01
	Raffinose	89.06	84.84	0.27	0.76	1.60	−0.32	0.81	2.08
	Stachyose	114.54	101.25	−0.97	−0.57	−0.18	−0.94	−0.48	−0.11
	Verbascose	86.06	86.18	−0.99	−0.98	−0.96	−0.99	−0.98	−0.94
	Glucose	118.12	80.41	−0.99	−0.99	−0.96	−1.00	−0.99	−0.95
	Maltose	112.80	109.89	−0.99	−0.95	−0.95	−0.99	−0.98	−0.97
Sugar Alcohols	Tetraols	88.90	73.78	−0.99	−0.99	−0.96	−0.99	−0.97	−0.93
	Pentitols	115.12	76.44	−0.99	−0.96	−0.90	−0.98	−0.95	−0.89
	Sorbitol	82.80	112.50	−0.87	−0.71	−0.50	−0.85	−0.73	−0.54
	Inositol	114.53	106.55	−0.95	−0.59	−0.49	−0.99	−0.79	−0.69
	Chiro-Inositol	104.99	95.55	−0.97	−0.30	0.21	−0.93	−0.55	−0.26
	Pinitol	107.46	101.65	−0.42	0.53	1.57	−0.36	1.22	2.62
	Galactinol	115.34	111.14	−0.07	0.34	1.19	−0.67	0.12	0.73
	Hexitols	93.73	113.30	−0.97	−0.89	−0.82	−0.95	−0.90	−0.84

RE: recovery, ME: matrix effect. * Soluble sugars and sugar alcohol concentration added to soybean seed extract.

**Table 3:** Sensitivity and linearity of GC–MS quantification method for hemicellulose components.

Carbohydrates	RT (min)	R^2^	LOD (ng µL^−1^)	LOQ (ng µL^−1^)
Rhamnose	10.20	0.9982	0.54	1.79
Fucose	10.49	0.9978	0.39	1.29
Arabinose	11.75	0.9949	0.30	1.10
Xylose	12.80	0.9977	0.43	1.43
Mannose	14.48	0.9981	0.77	2.56
Galactose	15.09	0.9990	0.90	3.00
Glucose	15.79	0.9963	0.92	3.06

Limits of detection (LOD) and limits of quantification (LOQ) were obtained based on a signal-to-noise ratio of 3:1 and 10:1, respectively. The linearity range was 3.33–333.33 ng µL^−1^ for all the sugars. RT: retention time, R^2^: correlation coefficient. An injection volume of 1 µL was considered.

**Table 4:** Recovery (%), matrix effect (%), intra- and inter-day accuracy (%) of the matrix polysaccharide.

			Accuracy (%)					
			Intra-Day Assay (n = 4)			Inter-Day Assay (n = 12)		
Carbo-hydrates	RE (%)	ME (%)	25 µg µL^−1^ *	50 µg µL^−1^ *	100 µg µL^−1^ *	25 µg µL^−1^ *	50 µg µL^−1^ *	100 µg µL^−1^ *
Rhamnose	108.84	90.72	2.13	7.52	14.12	2.87	7.62	14.88
Fucose	107.04	92.57	2.25	6.86	12.71	2.64	6.76	13.30
Arabinose	108.98	103.13	2.89	5.58	10.82	2.62	5.32	11.84
Xylose	104.33	100.05	2.21	6.65	12.09	2.79	6.02	12.53
Mannose	108.02	103.07	2.13	6.03	13.86	1.89	5.08	13.17
Galactose	108.06	108.64	1.06	3.87	11.94	1.53	4.24	12.52
Glucose	104.200	92.09	1.77	5.51	12.54	2.01	5.50	12.45

RE: recovery, ME: matrix effect. * Monosaccharide concentration added to soybean seed extract.

**Table 5:** Sensitivity and accuracy of anthrone quantification method for cellulose-derived glucose.

	Theoretical ng µL^−1^ Glucose	Measured ng µL^−1^ Glucose	Standard Deviation	RME (%)	LOD ng µL^−1^	LOQ ng µL^−1^	Equation R^2^
Day 1 n = 4	6.67	7.15	0.036	7.23	2.62	8.72	y = 0.032x + 0.0260 R^2^ = 0.9969
	13.33	13.11	0.055	−1.70			
	20.00	19.73	0.060	−1.35			
	26.67	25.96	0.051	−2.67			
	33.33	34.06	0.072	2.17			
Day 2 n = 4	6.67	7.41	0.032	11.09	1.03	3.45	y = 0.0312x + 0.0326 R^2^ = 0.9966
	13.33	12.58	0.029	−5.67			
	20.00	19.49	0.025	−2.55			
	26.67	27.00	0.065	1.23			
	33.33	33.53	0.078	0.59			
Day 3 n = 4	6.67	7.47	0.014	12.11	3.00	9.99	y = 0.0311x + 0.0339 R^2^ = 0.9920
	13.33	13.22	0.043	−0.83			
	20.00	18.43	0.014	−7.87			
	26.67	26.92	0.015	0.94			
	33.33	33.96	0.072	1.88			
Average n = 12	6.67	7.34	0.014	10.12	2.22	7.40	y = 0.0314x + 0.0225 R^2^ = 0.9967
	13.33	12.97	0.043	−2.72			
	20.00	19.22	0.014	−3.90			
	26.67	26.62	0.015	−0.19			
	33.33	33.85	0.072	1.55			

Limits of detection (LOD) and limits of quantification (LOQ) were defined as three and ten times the ratio between the standard deviation of y-axis intercept and the slope of the calibration curve, respectively. RME: relative mean error.

**Table 6:** Features that present significant differences between genotypes in each location (ANOVA, *p* < 0.05, n = 3)**.** For some of these features, a Fisher’s least significant difference (LSD) test was performed and the results are shown in Figure 2 and Figure 3. Glucose.1: glucose from matrix polysaccharide, TSS: total soluble sugars, TSA: total sugar alcohols, THC: hemicellulose components.

North Carolina		Arkansas	
Feature	*p*-value	Feature	*p*-value
Oil	3.59E−07	Sucrose	0.0002
Xylose	0.0029	Oil	0.0009
Protein	0.0035	Maltose	0.0049
Raffinose	0.0085	Raffinose	0.0073
THC	0.0166	Galactinol	0.0098
Pinitol	0.0358	Pentitols	0.0104
Glucose.1	0.0371	Inositol	0.0110
Galactose	0.0388	Protein	0.0152
TSA	0.0489	TSS	0.0201
Fucose	0.0489	Galactose	0.0232

## Supplementary Materials

The following are available online. Figure S1: Examples of LC–MS/MS chromatograms for each soluble sugar and sugar alcohol obtained using the external standard mix; Figure S2: Example of GC–MS chromatogram obtained using the external standard mix; Figure S3: Principal component analysis of the carbohydrate composition in eight soybean genotypes cultivated in two different locations (Arkansas and North Carolina); Figure S4: PCA biplots of PC1 vs. PC2 and PC1 vs. PC3, showing simultaneously the component loadings and scores; Table S1: Evaluation of the best procedure to remove moisture from seeds; Table S2. Principal component loadings; Table S3: Soluble sugar content of eight soybean genotypes cultivated in two different locations (Arkansas and North Carolina); Table S4: Sugar alcohol content of eight soybean genotypes cultivated in two different locations (Arkansas and North Carolina); Table S5: Hemicellulose content of eight soybean genotypes cultivated in two different locations (Arkansas and North Carolina); Table S6: Crystalline cellulose, oil, and protein content of eight soybean genotypes cultivated in two different locations (Arkansas and North Carolina).
