# Supplementary material for: Workflow for the Quantification of Soluble and Insoluble Carbohydrates in Soybean Seed
Source: Molecules. 2020 Aug 21;25(17):3806. doi: 10.3390/molecules25173806 (PMC7504011; doi:10.3390/molecules25173806)
Supplement: Supplementary file 1 [file molecules-25-03806-s001.pdf]

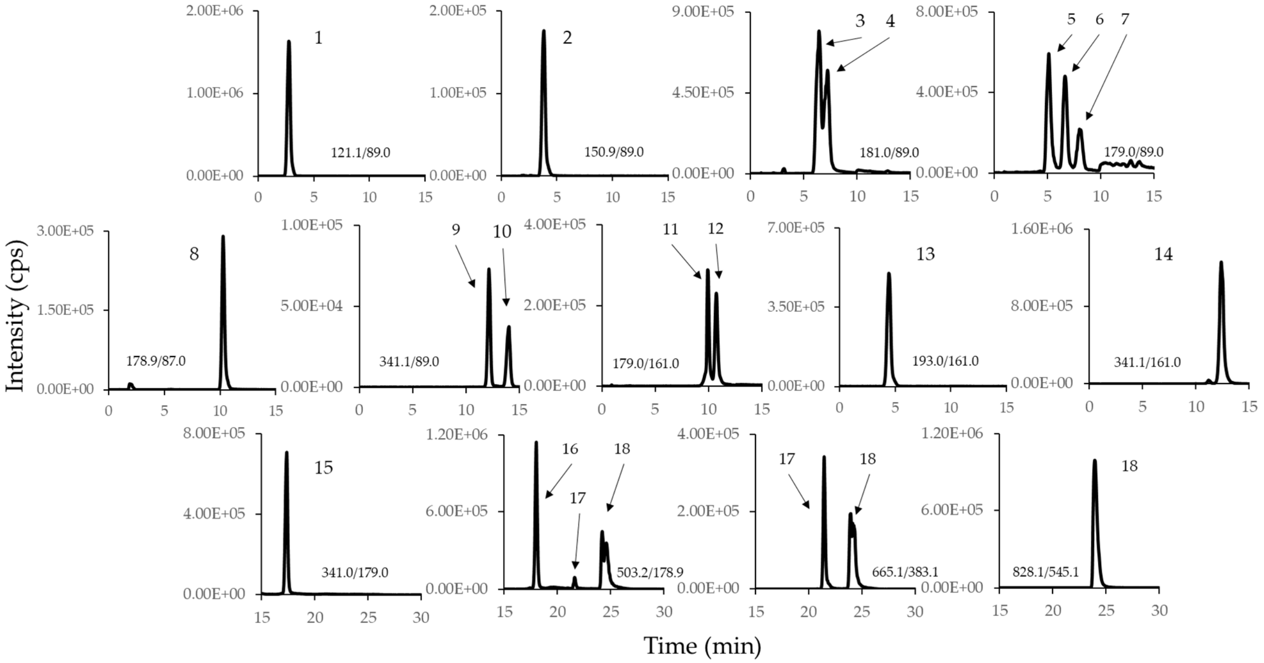

Figure S1. Examples of LC–MS/MS chromatograms for each soluble sugar and sugar alcohol obtained using the external standard mix. In addition to the metabolites described in Table 1, the mix contained Mannose, Trehalose and *Scyllo*-Inositol. These compounds were not further analyzed, as they were not present in soybean samples. In each chromatogram, the corresponding parent/daughter ion transition is shown and the time scale was adapted for better visualization of the peaks. Isomers, such as Sorbitol/Hexitols, Fructose/Glucose, Sucrose/Trehalose and *Chiro*-Inositol/*Scyllo*-Inositol, are quantified in the same transition, as they share the same molecular weight. On the other hand, it is possible to observed more than one peak at a given transition when the ionization source of the MS cleaves part of the structure of a metabolite. For example, in the Raffinose's transition (503.2/178.9) it is possible to observe the peaks of Stachyose and Verbascose at their specific retention times, as well. Verbascose is a pentasaccharide and Stachyose a tetrasaccharide, and, in the source, a portion of these oligosaccharides loose two and one hexose moiety, respectively, resulting in a structure with the molecular weight of a trisaccharide. This explains why they are seen at the same transitions as the trisaccharide Raffinose. The same phenomenum occurs for a proportion of Verbascose that looses a hexose in the source, and therefore can be detected in the same transition than the tetrasaccharide Stachyose. However, the quantification of Stachyose and Verbascose was performed at their specific transitions, where better sensitivity was observed. 1: Tetraols, 2: Pentitols, 3: Sorbitol, 4: Hexitols, 5: Fructose, 6: Mannose, 7: Glucose, 8: Inositol, 9: Sucrose, 10: Trehalose, 11: *Chiro*-Inositol, 12: *Scyllo*-Inositol, 13: Pinitol, 14: Maltose, 15: Galactinol, 16: Raffinose, 17: Stachyose and 18: Verbascose.

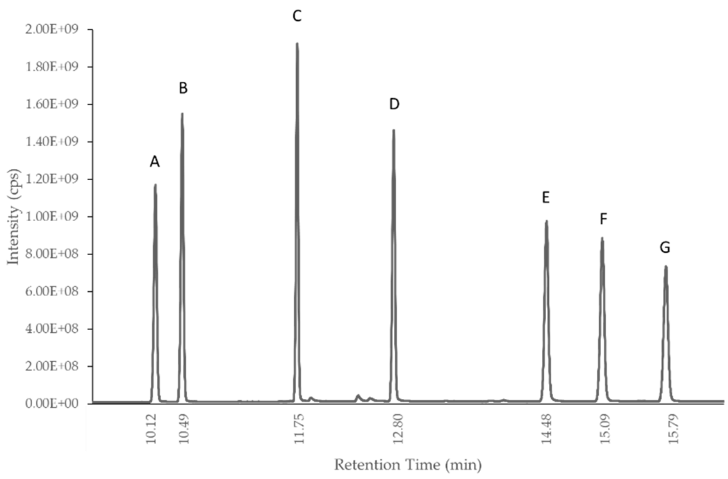

Figure S2. Example of GC-MS chromatogram obtained using the external standard mix. Each peak corresponds to a monosaccharide standard. A: rhamnose, B: fucose, C: arabinose, D: xylose, E: mannose, F: galactose and G: glucose.

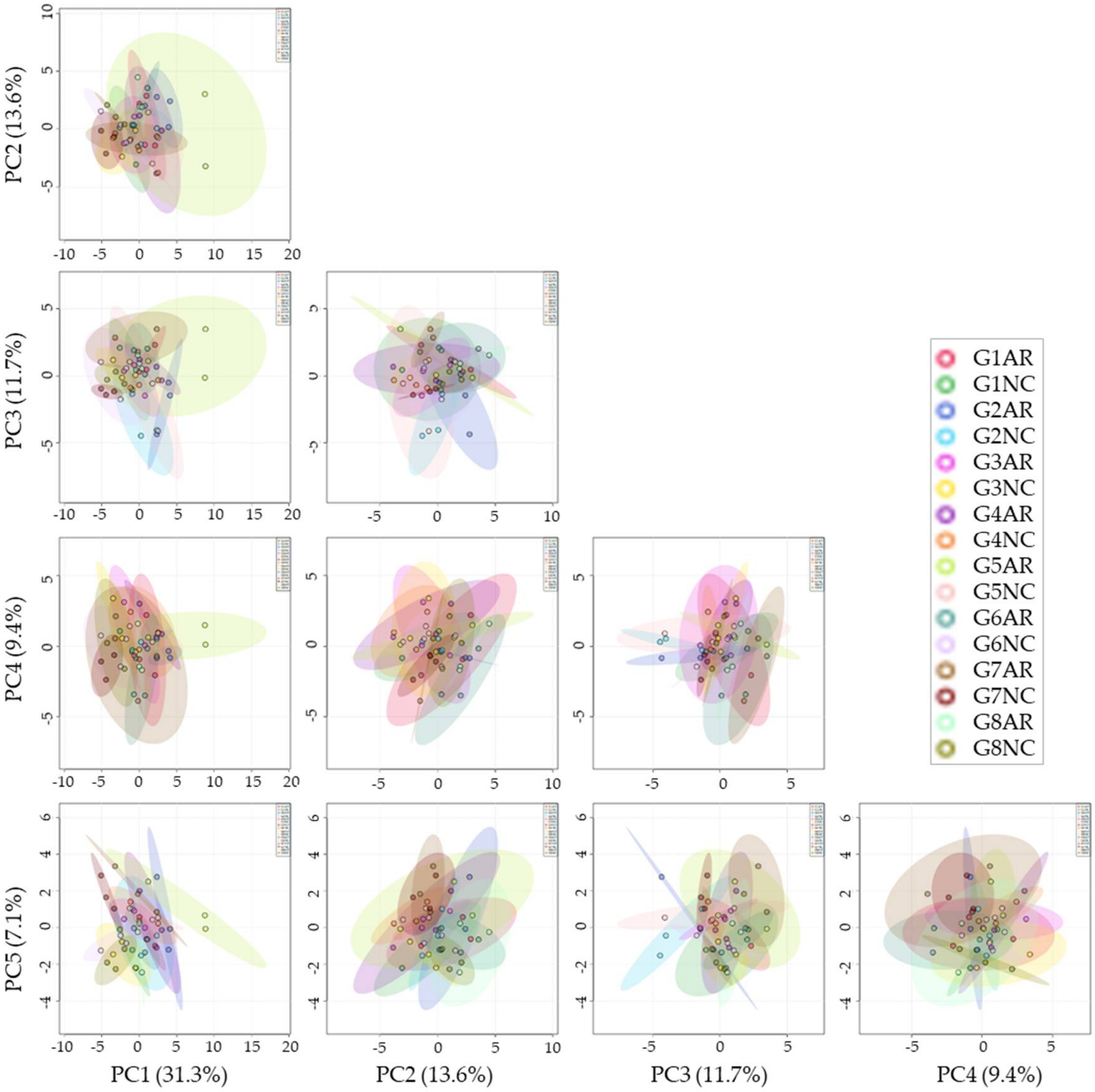

Figure S3. Principal component analysis of the carbohydrate composition in eight soybean genotypes cultivated in two different locations (Arkansas and North Carolina). The first five components are displayed in a two-dimensional combination, and the explained variances are detailed in parentheses.

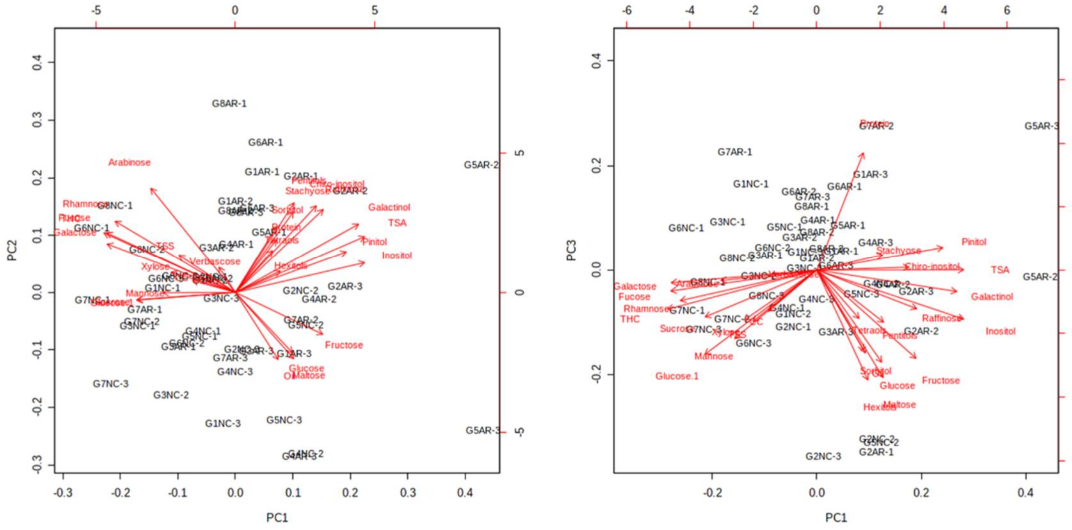

Figure S4. PCA biplots of PC1 vs. PC2 and PC1 vs. PC3, showing simultaneously the component loadings and scores.

Table S1. Evaluation of the best procedure to remove moisture from seeds. In the first procedure, oriented to evaluate between using whole seed or the ground seed (sample 1 to 6), 24 mature soybean seeds of the same genotype were use, four seeds for each replicate, and three biological replicates per seed treatment. The second procedure, focused on the selection of the best drying method (sample 7 to 12), used six mature soybean seeds of the same genotype that were ground, homogenized, and divided into six parts. Three biological replicates per drying method were used. Significant differences are indicated by different letters as superscript (ANOVA, Fisher LSD, $p<0.05$, $n=3$).

Sample	Seed treatment	Drying method	Fresh weight (mg)	Dry weight (mg)	Moisture removed (%)	Average moisture removed (%)
**1**	Whole seed	Lyophilizer	561.47	540.87	3.67	
**2**	Whole seed	Lyophilizer	589.86	567.94	3.72	3.68 ^{a}
**3**	Whole seed	Lyophilizer	580.86	559.57	3.67	
**4**	Ground seed	Lyophilizer	573.95	538.73	6.14	
**5**	Ground seed	Lyophilizer	571.64	536.41	6.16	6.19 ^{b}
**6**	Ground seed	Lyophilizer	572.48	536.56	6.27	
**7**	Ground seed	Oven	101.56	92.63	8.79	
**8**	Ground seed	Oven	100.82	93.10	7.66	8.52 ^{c}
**9**	Ground seed	Oven	109.02	99.08	9.12	
**10**	Ground seed	Lyophilizer	115.32	107.46	6.82	
**11**	Ground seed	Lyophilizer	101.36	95.68	5.60	6.36 ^{b}
**12**	Ground seed	Lyophilizer	101.78	95.00	6.66	

Table S2. Principal component loadings

	PC1	PC2	PC3	PC4	PC5
**TSS**	-0.1226	0.1236	-0.1613	0.2976	-0.3067
**Fructose**	0.1903	-0.1386	-0.2776	-0.1237	-0.1838
**Sucrose**	-0.2142	-0.0242	-0.1475	0.1978	-0.2756
**Raffinose**	0.1915	0.2753	-0.1217	-0.0144	-0.2549
**Stachyose**	0.1262	0.2673	0.0484	0.2467	0.0480
**Verbascose**	-0.0356	0.0835	-0.0109	**0.3660**	0.0828
**Glucose**	0.1243	-0.1993	-0.2895	-0.1634	-0.2792
**Maltose**	0.1273	-0.2194	**-0.3379**	0.1121	0.0312
**TSA**	**0.2817**	0.1856	0.0006	-0.0307	0.1990
**Tetraols**	0.0808	0.1389	-0.1521	0.2190	0.2361
**Pentitols**	0.1280	**0.2974**	-0.1640	-0.1108	-0.1809
**Sorbitol**	0.0900	0.2190	-0.2523	0.1309	0.0962
**Inositol**	**0.2817**	0.0990	-0.1542	-0.1793	-0.0452
**Chiro-inositol**	0.1765	**0.2875**	0.0091	-0.0947	-0.2483
**Pinitol**	**0.2416**	0.1346	0.0714	0.0221	0.3060
**Galactinol**	**0.2685**	0.2268	-0.0677	-0.0791	-0.0824
**Hexitols**	0.0979	0.0713	**-0.3452**	0.1253	0.2583
**THC**	**-0.2863**	0.1977	-0.1231	-0.1011	0.0255
**Rhamnose**	**-0.2618**	0.2363	-0.0963	0.0503	0.1346
**Fucose**	**-0.2802**	0.1981	-0.0660	0.0861	-0.0573
**Arabinose**	-0.1839	**0.3451**	-0.0332	-0.0577	0.0433
**Xylose**	-0.1393	0.0676	-0.1571	-0.2933	**0.4109**
**Mannose**	-0.1576	-0.0015	-0.2157	**-0.3389**	0.0343
**Galactose**	**-0.2792**	0.1610	-0.0400	0.0381	-0.1567
**Glucose.1**	-0.2146	-0.0259	-0.2653	-0.1165	0.1440
**CC**	-0.0930	0.0389	-0.1299	**-0.3934**	0.0264
**Protein**	0.0893	0.1746	**0.3684**	-0.1534	0.0344
**Oil**	0.0934	-0.2218	-0.2605	0.2516	0.1843

The values larger than 80% of maximum loading for a given PC are shown in bold. TSS: total soluble sugars, TSA: total sugar alcohols, THC: hemicellulose components, Glucose.1: glucose from matrix polysaccharide, and CC: Crystalline cellulose.

Table S3. Soluble sugar content of eight soybean genotypes cultivated in two different locations (Arkansas and North Carolina). Data shown represents the average concentration of three biological replicates in mg per g dry weight (mg.g^{-1}) ± standard deviation. Significant differences between genotypes per location are indicated by different letters (ANOVA, Fisher LSD, $p<0.05$, $n=3$).

Location	Genotype		Soluble sugar content (mg.g^{-1})							
			Fructose	Sucrose	Raffinose	Stachyose	Verbascose	Glucose	Maltose	Total soluble sugar
Arkansas	G1	R15-5695	0.08 ± 0.09^{a}	69.49 ± 16.14^{a}	5.29 ± 1.81^{a}	17.40 ± 2.90^{a}	0.24 ± 0.09^{a}	0.18 ± 0.23^{a}	0.00 ± 0.00^{a}	92.68 ± 19.20^{ab}
	G2	N16-502	0.19 ± 0.04^{a}	80.03 ± 4.71^{bc}	11.04 ± 0.94^{b}	23.02 ± 2.74^{a}	0.28 ± 0.09^{a}	0.38 ± 0.18^{a}	0.01 ± 0.00^{bc}	114.96 ± 6.72^{a}
	G3	R15-7171	0.08 ± 0.03^{a}	106.39 ± 6.85^{d}	6.17 ± 0.56^{a}	27.12 ± 4.10^{a}	0.38 ± 0.11^{a}	0.20 ± 0.18^{a}	0.01 ± 0.01^{bc}	140.36 ± 10.03^{c}
	G4	N16-1119	0.20 ± 0.14^{a}	96.36 ± 1.04^{a}	6.11 ± 1.35^{a}	25.70 ± 2.83^{a}	0.45 ± 0.18^{a}	0.39 ± 0.30^{a}	0.02 ± 0.01^{b}	129.24 ± 4.52^{b}
	G5	N16-1091	0.26 ± 0.24^{a}	60.30 ± 7.93^{e}	11.36 ± 4.56^{b}	29.11 ± 11.39^{a}	0.61 ± 0.26^{a}	0.42 ± 0.38^{a}	0.01 ± 0.00^{c}	102.06 ± 13.98^{d}
	G6	AG 56X8	0.14 ± 0.08^{a}	84.68 ± 5.34^{b}	9.96 ± 1.70^{b}	20.12 ± 6.19^{a}	0.40 ± 0.18^{a}	0.29 ± 0.19^{a}	0.00 ± 0.00^{a}	115.59 ± 9.76^{a}
	G7	AG 59X7	0.08 ± 0.09^{a}	75.21 ± 8.11^{c}	5.28 ± 2.86^{a}	20.18 ± 2.41^{a}	0.31 ± 0.12^{a}	0.16 ± 0.17^{a}	0.00 ± 0.00^{a}	101.23 ± 10.59^{d}
	G8	NC-Dunphy	0.09 ± 0.08^{a}	82.26 ± 8.22^{bc}	11.07 ± 1.61^{b}	26.22 ± 4.80^{a}	0.38 ± 0.11^{a}	0.23 ± 0.24^{a}	0.00 ± 0.00^{a}	120.25 ± 13.63^{ab}
North Carolina	G1	R15-5695	0.24 ± 0.17^{a}	104.04 ± 9.93^{a}	5.69 ± 0.86^{ab}	17.70 ± 2.50^{a}	0.42 ± 0.09^{a}	0.50 ± 0.30^{a}	0.00 ± 0.00^{a}	128.60 ± 12.26^{a}
	G2	N16-502	0.54 ± 0.53^{a}	94.36 ± 13.58^{a}	8.62 ± 1.86^{c}	21.00 ± 0.56^{a}	0.46 ± 0.10^{a}	0.86 ± 0.61^{a}	0.03 ± 0.02^{b}	125.86 ± 16.60^{a}
	G3	R15-7171	0.08 ± 0.04^{a}	108.79 ± 9.11^{a}	6.62 ± 1.75^{ad}	19.04 ± 2.88^{a}	0.48 ± 0.07^{a}	0.26 ± 0.07^{a}	0.01 ± 0.01^{a}	135.28 ± 7.24^{a}
	G4	N16-1119	0.23 ± 0.16^{a}	85.26 ± 4.11^{a}	6.41 ± 1.12^{ad}	18.32 ± 1.20^{a}	0.44 ± 0.08^{a}	0.44 ± 0.26^{a}	0.02 ± 0.00^{c}	111.10 ± 4.45^{a}
	G5	N16-1091	0.15 ± 0.25^{a}	64.95 ± 9.80^{a}	6.22 ± 1.14^{ce}	13.28 ± 2.13^{a}	0.39 ± 0.14^{a}	0.29 ± 0.30^{a}	0.01 ± 0.01^{a}	85.30 ± 12.55^{a}
	G6	AG 56X8	0.07 ± 0.04^{a}	95.93 ± 6.09^{a}	7.32 ± 1.24^{de}	18.86 ± 1.68^{a}	0.51 ± 0.14^{a}	0.22 ± 0.09^{a}	0.00 ± 0.00^{a}	122.91 ± 6.05^{a}
	G7	AG 59X7	0.06 ± 0.03^{a}	96.41 ± 2.90^{a}	4.84 ± 2.51^{b}	15.44 ± 7.87^{a}	0.41 ± 0.21^{a}	0.23 ± 0.12^{a}	0.00 ± 0.00^{a}	117.40 ± 58.86^{a}
	G8	NC-Dunphy	0.06 ± 0.03^{a}	105.91 ± 17.07^{a}	8.49 ± 0.42^{c}	20.29 ± 2.17^{a}	0.43 ± 0.17^{a}	0.26 ± 0.13^{a}	0.00 ± 0.00^{a}	135.44 ± 18.68^{a}

Table S4. Sugar alcohol content of eight soybean genotypes cultivated in two different locations (Arkansas and North Carolina). Data shown represents the average concentration of three biological replicates in mg per g dry weight (mg.g^{-1}) ± standard deviation. Significant differences between genotypes per location are indicated by different letters (ANOVA, Fisher LSD, $p<0.05$, n=3).

Location		Genotype	Sugar alcohol content (mg.g^{-1})								
			**Tetraols**	**Pentitols**	**Sorbitol**	**Inositol**	**Chiro-inositol**	**Pinitol**	**Galactinol**	**Hexitols**	**Total sugar alcohols**
**Arkansas**	G1	R15-5695	0.01 ± 0.00^{a}	0.04 ± 0.03^{ade}	0.02 ± 0.01^{a}	0.27 ± 0.04^{acd}	0.19 ± 0.06^{a}	2.75 ± 0.62^{a}	0.62 ± 0.10^{a}	0.02 ± 0.01^{a}	3.93 ± 0.74^{a}
	G2	N16-502	0.02 ± 0.01^{a}	0.08 ± 0.03^{c}	0.09 ± 0.09^{a}	1.00 ± 0.17^{b}	0.26 ± 0.07^{a}	4.18 ± 0.70^{a}	1.20 ± 0.14^{b}	0.05 ± 0.03^{a}	6.89 ± 0.90^{a}
	G3	R15-7171	0.01 ± 0.00^{a}	0.03 ± 0.01^{b}	0.02 ± 0.00^{a}	0.27 ± 0.11^{c}	0.16 ± 0.05^{a}	3.91 ± 0.72^{a}	0.56 ± 0.12^{c}	0.04 ± 0.01^{a}	5.00 ± 0.91^{a}
	G4	N16-1119	0.02 ± 0.01^{a}	0.02 ± 0.00^{b}	0.02 ± 0.00^{a}	0.39 ± 0.10^{acd}	0.17 ± 0.05^{a}	4.12 ± 0.82^{a}	0.81 ± 0.17^{a}	0.04 ± 0.01^{a}	5.59 ± 0.99^{a}
	G5	N16-1091	0.02 ± 0.01^{a}	0.05 ± 0.03^{d}	0.03 ± 0.01^{a}	0.83 ± 0.46^{d}	0.28 ± 0.10^{a}	5.91 ± 2.22^{a}	1.12 ± 0.40^{b}	0.05 ± 0.02^{a}	8.28 ± 3.20^{a}
	G6	AG 56X8	0.01 ± 0.00^{a}	0.07 ± 0.03^{ce}	0.02 ± 0.01^{a}	0.46 ± 0.09^{bc}	0.22 ± 0.01^{a}	3.87 ± 0.72^{a}	0.86 ± 0.17^{a}	0.03 ± 0.00^{a}	5.54 ± 0.92^{a}
	G7	AG 59X7	0.01 ± 0.00^{a}	0.02 ± 0.01^{b}	0.02 ± 0.00^{a}	0.30 ± 0.16^{acd}	0.15 ± 0.05^{a}	3.69 ± 0.05^{a}	0.54 ± 0.28^{c}	0.04 ± 0.00^{a}	4.77 ± 0.48^{a}
	G8	NC-Dunphy	0.01 ± 0.00^{a}	0.05 ± 0.02^{ade}	0.03 ± 0.01^{a}	0.45 ± 0.14^{acd}	0.25 ± 0.07^{a}	3.16 ± 0.72^{a}	0.91 ± 0.10^{a}	0.04 ± 0.01^{a}	4.89 ± 0.48^{a}
**North Carolina**	G1	R15-5695	0.01 ± 0.00^{a}	0.03 ± 0.01^{a}	0.02 ± 0.00^{a}	0.22 ± 0.09^{a}	0.24 ± 0.05^{a}	2.82 ± 0.53^{a}	0.43 ± 0.11^{a}	0.03 ± 0.00^{a}	3.80 ± 0.61^{a}
	G2	N16-502	0.01 ± 0.00^{a}	0.06 ± 0.01^{a}	0.05 ± 0.01^{a}	0.65 ± 0.36^{a}	0.17 ± 0.02^{a}	2.96 ± 0.66^{a}	0.70 ± 0.15^{a}	0.05 ± 0.01^{a}	4.66 ± 1.09^{a}
	G3	R15-7171	0.01 ± 0.00^{a}	0.04 ± 0.04^{a}	0.03 ± 0.02^{a}	0.21 ± 0.10^{a}	0.17 ± 0.09^{a}	2.35 ± 0.33^{b}	0.49 ± 0.14^{a}	0.03 ± 0.01^{a}	3.33 ± 0.65^{a}
	G4	N16-1119	0.05 ± 0.07^{a}	0.02 ± 0.01^{a}	0.02 ± 0.01^{a}	0.30 ± 0.09^{a}	0.15 ± 0.03^{a}	3.08 ± 0.72^{a}	0.56 ± 0.06^{a}	0.04 ± 0.00^{a}	4.22 ± 0.75^{a}
	G5	N16-1091	0.01 ± 0.00^{a}	0.03 ± 0.02^{a}	0.03 ± 0.04^{a}	0.23 ± 0.14^{a}	0.13 ± 0.05^{a}	2.72 ± 0.36^{c}	0.43 ± 0.11^{a}	0.04 ± 0.02^{a}	3.62 ± 0.67^{a}
	G6	AG 56X8	0.01 ± 0.00^{a}	0.04 ± 0.01^{a}	0.03 ± 0.00^{a}	0.18 ± 0.07^{a}	0.16 ± 0.01^{a}	2.26 ± 0.14^{b}	0.53 ± 0.11^{a}	0.03 ± 0.01^{a}	3.23 ± 0.32^{a}
	G7	AG 59X7	0.01 ± 0.00^{a}	0.02 ± 0.01^{a}	0.02 ± 0.01^{a}	0.16 ± 0.09^{a}	0.12 ± 0.06^{a}	2.94 ± 1.57^{a}	0.42 ± 0.21^{a}	0.05 ± 0.02^{a}	3.74 ± 1.93^{a}
	G8	NC-Dunphy	0.01 ± 0.00^{a}	0.03 ± 0.02^{a}	0.02 ± 0.01^{a}	0.29 ± 0.24^{a}	0.24 ± 0.06^{a}	2.23 ± 0.26^{b}	0.54 ± 0.09^{a}	0.04 ± 0.01^{a}	3.39 ± 0.24^{a}

Table S5. Hemicellulose content of eight soybean genotypes cultivated in two different locations (Arkansas and North Carolina). Data shown represents the average concentration of three biological replicates in mg per g dry weight (mg.g^{-1}) ± standard deviation. Significant differences between genotypes per location are indicated by different letters (ANOVA, Fisher LSD, $p<0.05$, n=3).

Location	Genotype		Hemicellulose content (mg.g^{-1})							
			**Rhamnose**	**Fucose**	**Arabinose**	**Xylose**	**Mannose**	**Galactose**	**Glucose**	**Total hemicellulose**
**Arkansas**	G1	R15-5695	3.01 ± 0.53^{a}	1.70 ± 0.20^{a}	11.27 ± 2.03^{a}	6.74 ± 1.87^{a}	8.95 ± 1.77^{a}	19.72 ± 1.92^{abd}	3.10 ± 0.54^{a}	54.49 ± 5.05^{a}
	G2	N16-502	3.82 ± 0.51^{a}	2.20 ± 0.23^{a}	14.92 ± 1.22^{a}	9.83 ± 2.08^{a}	12.99 ± 0.47^{a}	25.56 ± 0.82^{ab}	4.22 ± 0.80^{a}	73.55 ± 3.78^{a}
	G3	R15-7171	4.03 ± 0.38^{a}	2.42 ± 0.36^{a}	13.71 ± 0.24^{a}	9.80 ± 1.11^{a}	12.03 ± 1.56^{a}	26.51 ± 0.53^{a}	4.98 ± 0.27^{a}	73.48 ± 2.34^{a}
	G4	N16-1119	3.67 ± 0.60^{a}	2.22 ± 0.27^{a}	13.42 ± 1.29^{a}	8.98 ± 0.68^{a}	11.05 ± 0.42^{a}	24.56 ± 1.63^{bc}	4.10 ± 0.35^{a}	68.00 ± 4.32^{a}
	G5	N16-1091	3.31 ± 0.82^{a}	1.99 ± 0.54^{a}	11.75 ± 2.92^{a}	8.70 ± 2.10^{a}	10.69 ± 1.01^{a}	22.92 ± 4.73^{c}	3.51 ± 0.52^{a}	62.88 ± 12.55^{a}
	G6	AG 56X8	3.94 ± 0.23^{a}	2.55 ± 0.24^{a}	15.25 ± 0.47^{a}	10.67 ± 0.96^{a}	12.70 ± 1.30^{a}	27.78 ± 0.94^{d}	4.32 ± 0.52^{a}	77.20 ± 0.94^{a}
	G7	AG 59X7	3.89 ± 0.74^{a}	2.26 ± 0.29^{a}	13.66 ± 1.30^{a}	10.90 ± 1.21^{a}	12.61 ± 1.84^{a}	26.35 ± 2.18^{abd}	4.14 ± 0.51^{a}	73.82 ± 7.37^{a}
	G8	NC -Dunphy	4.00 ± 0.24^{a}	2.68 ± 0.10^{a}	15.50 ± 1.06^{a}	9.33 ± 0.75^{a}	12.11 ± 0.86^{a}	31.51 ± 1.03^{e}	4.33 ± 0.44^{a}	79.45 ± 0.65^{a}
**North Carolina**	G1	R15-5695	4.00 ± 0.40^{a}	2.45 ± 0.31^{abc}	13.87 ± 1.62^{a}	9.23 ± 1.16^{a}	12.39 ± 1.22^{a}	29.86 ± 2.97^{ab}	4.43 ± 0.52^{a}	76.25 ± 4.98^{a}
	G2	N16-502	4.01 ± 0.17^{a}	2.38 ± 0.10^{b}	13.91 ± 0.45^{a}	9.84 ± 0.58^{a}	13.66 ± 0.76^{a}	28.94 ± 2.39^{ab}	4.84 ± 0.15^{b}	77.57 ± 4.19^{a}
	G3	R15-7171	4.00 ± 0.23^{a}	2.67 ± 0.34^{c}	12.75 ± 0.72^{a}	8.31 ± 0.58^{b}	12.89 ± 1.26^{a}	30.55 ± 3.29^{abd}	4.61 ± 0.69^{ab}	75.77 ± 2.18^{a}
	G4	N16-1119	3.62 ± 0.84^{a}	2.35 ± 0.53^{b}	13.03 ± 2.15^{a}	9.80 ± 0.78^{a}	11.83 ± 1.36^{a}	28.36 ± 2.37^{bc}	4.59 ± 0.57^{ab}	73.58 ± 5.56^{ab}
	G5	N16-1091	2.76 ± 0.28^{a}	1.79 ± 0.29^{abc}	8.96 ± 1.74^{a}	6.94 ± 0.76^{a}	9.54 ± 0.54^{a}	19.36 ± 3.25^{c}	3.43 ± 0.27^{ab}	52.78 ± 5.83^{b}
	G6	AG 56X8	4.27 ± 0.46^{a}	3.07 ± 0.42^{de}	14.73 ± 1.83^{a}	9.65 ± 1.07^{a}	13.41 ± 0.69^{a}	33.22 ± 5.27^{d}	4.66 ± 0.34^{ab}	83.03 ± 8.14^{c}
	G7	AG 59X7	4.66 ± 2.36^{a}	2.81 ± 1.41^{d}	14.71 ± 7.37^{a}	12.9 ± 6.52^{c}	14.37 ± 7.20^{a}	31.36 ± 15.74^{a}	5.89 ± 2.97^{c}	86.7 ± 43.48^{c}
	G8	NC-Dunphy	4.40 ± 0.39^{a}	3.14 ± 0.16^{e}	15.28 ± 1.38^{a}	9.16 ± 1.14^{a}	12.55 ± 0.11^{a}	35.60 ± 2.65^{e}	4.98 ± 0.13^{c}	85.12 ± 5.31^{c}

Table S6. Crystalline cellulose, oil and protein content of eight soybean genotypes cultivated in two different locations (Arkansas and North Carolina). Data shown represents the average concentration of three biological replicates in percentage ± standard deviation. Significant differences between genotypes per location are indicated by different letters (ANOVA, Fisher LSD, $p<0.05$, $n=3$).

Location	Genotype		Crystalline cellulose (%)	Protein (%)	Oil (%)
Arkansas	G1	R15-5695	3.41 ± 1.14^{a}	31.26 ± 1.45^{ab}	18.06 ± 1.01^{ac}
	G2	N16-502	4.24 ± 0.53^{a}	39.37 ± 1.59^{cd}	25.16 ± 0.81^{bd}
	G3	R15-7171	5.12 ± 0.45^{a}	39.50 ± 2.93^{cde}	24.92 ± 1.49^{bde}
	G4	N16-1119	3.84 ± 0.15^{a}	40.08 ± 1.80^{de}	24.75 ± 0.83^{cd}
	G5	N16-1091	3.46 ± 0.72^{a}	43.46 ± 0.61^{f}	24.40 ± 0.63^{ce}
	G6	AG 56X8	5.14 ± 1.08^{a}	43.14 ± 0.43^{f}	22.01 ± 0.46^{f}
	G7	AG 59X7	4.31 ± 0.51^{a}	42.56 ± 0.26^{af}	23.03 ± 0.21^{g}
	G8	NC-Dunphy	4.43 ± 0.49^{a}	40.75 ± 0.12^{be}	22.66 ± 0.21^{fg}
North Carolina	G1	R15-5695	4.43 ± 0.67^{a}	41.42 ± 0.63^{a}	24.12 ± 0.44^{a}
	G2	N16-502	4.15 ± 0.21^{a}	37.18 ± 0.67^{b}	25.21 ± 0.58^{b}
	G3	R15-7171	3.93 ± 0.98^{a}	39.20 ± 1.21^{c}	23.71 ± 0.41^{c}
	G4	N16-1119	3.22 ± 0.39^{a}	38.06 ± 0.81^{b}	25.04 ± 0.30^{b}
	G5	N16-1091	3.12 ± 1.32^{a}	27.91 ± 1.95^{b}	19.66 ± 0.24^{d}
	G6	AG 56X8	4.67 ± 0.76^{a}	38.99 ± 1.01^{c}	22.97 ± 0.45^{e}
	G7	AG 59X7	5.22 ± 2.73^{a}	37.82 ± 18.91^{b}	24.11 ± 12.06^{a}
	G8	NC-Dunphy	4.26 ± 0.44^{a}	39.11 ± 0.97^{c}	22.85 ± 0.33^{e}
